# Core Competencies for Psychological Counselors: A Scoping Review

**DOI:** 10.3390/bs15020147

**Published:** 2025-01-29

**Authors:** Cheng Chen, Yandi Zhang, Qing Guo, Xuanyi Wang, Shulin Chen

**Affiliations:** Department of Psychology and Behavioral Sciences, Zhejiang University, Hangzhou 310058, China; chencheng_psy@zju.edu.cn (C.C.); 3200100592@zju.edu.cn (Y.Z.); 3200101143@zju.edu.cn (Q.G.)

**Keywords:** counselor, competency, scoping review, mental health, psychotherapy

## Abstract

Treatment provided by psychological counselors is a professional solution to prevailing mental health issues. Despite the presence of several narrative reviews, the counseling competencies have not been systematically examined based on empirical studies. Therefore, the present study aimed to comprehensively clarify the core counseling competencies using the scoping review method. Five databases were searched, including Web of Science, PsycINFO, PubMed, WANFANG, and CNKI. The articles were included or excluded based on strict criteria of having a specific focus on the general counseling competencies. The competencies were extracted using a three-stage coding process following the principles of the Grounded Theory method. Only 15 studies qualified and were subsequently coded to extract the relevant competencies. Through the three-level coding, 122 competencies were initially identified; then, 30 core competencies were obtained by merging similar items. Based on the attributes of the core competencies, four categories finally emerged: (1) attitude, (2) personality, (3) knowledge, and (4) skill and ability. The precise definition of each core competence was explicitly articulated and presented with references. The category of skill and ability emerged as the most frequently reported in the articles reviewed. The empirical research on counseling competencies was limited. The present study enumerated 30 core competencies for counselors and further extracted four categories from them. The findings contribute to the establishment of a compelling framework that facilitates a comprehensive understanding of the pivotal areas for counselors’ professional growth and development.

## 1. Introduction

Mental health is an integral part of general human health and well-being (WHO, 2022) and is essential for sustainable human development (Prince et al., 2007). However, mental disorders have been one of the primary contributors to the overall worldwide health burden (Moitra et al., 2023). For instance, the collective number of cases globally for depression and anxiety exceeded 970 million in 2019 (GBD 2019 Mental Disorders Collaborators, 2022). In the context of the high incidence of severe mental health issues, a significant portion of individuals in need of mental health care worldwide lack access to high-quality mental health services (Wainberg et al., 2017). Counseling provided by psychological counselors is a global professional solution to address prevalent mental health issues. This scoping review seeks to provide an integrative description of the core counseling competencies based on existing evidence, aiming to support the development of more effective psychotherapy practices.

### 1.1. Counseling Competency

Counseling competency essentially is a specified form of competency. Competency itself refers to a set of inherent qualities and acquired skills that contribute to individual and organizational performance. The concept of competency was initially introduced by McClelland (1973) as a means to supplant conventional intelligence tests. According to McClelland (1973), it is competency that leads to effective task execution rather than intelligence. Competency could be broadly divided into visible aspects (i.e., knowledge, skills, and behaviors that are relatively apparent characteristics of an individual) and hidden aspects (i.e., individual underlying attributes, such as traits, motives, attitudes, values, and self-image; Salman et al., 2020; Spencer & Spencer, 1993). Employee competencies exhibit a positive relationship with organizational performance (Mandourah et al., 2017; Osei & Ackah, 2015; Potnuru & Sahoo, 2016), which provides guidance for hiring employees. In conclusion, competency encompasses the essential characteristics and skills necessary for efficient and effective job performance. Specifically, counseling competency directly influences a counselor’s professional performance during the counseling process. When facing complex and diverse counseling scenarios, it provides a series of indispensable supports, enabling counseling work to be conducted efficiently and with high quality. To facilitate competent job performance among counselors, it is essential to clarify the core counseling competencies.

### 1.2. Dimensions of Counseling Competency

Prior research has presented a variety of viewpoints regarding counselor competencies. For instance, Jennings and Skovholt (1999) classified competencies into cognitive, emotional, and relational dimensions using interview-based methods. The cognitive dimension refers to the ability to engage in systematic and in-depth case conceptualization and make accurate diagnoses; the emotional dimension is mainly about emotional acceptance; and the relational dimension focuses on relationship-building and maintenance skills. Roe (2002) posited that professional competencies in psychotherapy consisted of knowledge, skills, attitudes, abilities, personality traits, and other characteristics. When developing a questionnaire on school counselors’ competency, Xie (2008) formulated a set of competencies across six dimensions: (1) professional personality traits, (2) self-growth traits, (3) solid professional knowledge base and influence, (4) self-improvement, (5) good professionalism and ethics, and (6) social personality traits. M. Wang et al. (2015) derived the core competencies for China’s counselors and therapists through expert seminars, including six dimensions: (1) professional attitudes and behaviors, (2) ethics and law; (3) clinical knowledge and skills; (4) science and research; (5) relationship building, multiculturalism, and Chinese culture; and (6) case management. Fan (2018) developed a framework delineating the essential knowledge and skills required for counselors. This framework comprises areas such as ethics of counseling, psychological assessment, mental health education, crisis identification, and intervention. Therefore, multiple studies have examined counselor competencies (Sperry & Sperry, 2023).

However, it should be noted that each of the existing models has certain limitations. For instance, the competency model proposed by Jennings and Skovholt (1999), while comprising three dimensions (cognitive, emotional, and relational), primarily emphasizes abilities or skills and overlooks counselor traits or personality—a limitation also found in other models. Additionally, some models incorporate an extensive number of dimensions, which can hinder their practical application and dissemination due to their complexity. Furthermore, certain dimensions within these models may overlap, potentially leading to redundancy and confusion in their interpretation and use.

As a result, most of these models have not been widely adopted, and no unanimous consensus has been reached regarding the composition of core competencies or their definitions. Therefore, a more comprehensive and holistic understanding is needed, and systematic reviews could serve as a valuable method for synthesizing existing evidence in a reliable manner.

### 1.3. The Need for Systematic Reviews on Counseling Competencies

Seldom have systematic reviews on counseling competencies been published. We conducted literature searches using the keywords including “competency”, “counselor”, and “review” in the Web of Science and PubMed databases in March 2024. Only a few narrative reviews concerning counseling competencies for counselors were identified. Furthermore, none of these reviews specifically outlined the essential competencies required for psychological counselors. Instead, they had other specified research foci, such as the necessity of cultural competencies to address the needs of diverse populations (Hays, 2008), the application of evidence-based practices such as motivational interviewing (Gill et al., 2020), training in a specific therapy (such as cognitive behavioral therapy (CBT); Henrich et al., 2023), the significance of clinical supervision (Falender et al., 2004), and ethical considerations and self-awareness in the therapeutic process (Lohani & Sharma, 2023). Therefore, there is a notable dearth of systematic reviews that specifically elucidate the definitions and categorizations of counseling competencies.

A scoping review is a type of systematic review. While traditional systematic reviews aim to answer specific research questions by synthesizing and analyzing primary studies, scoping reviews have a broader objective of mapping the existing literature on a particular topic, and identifying key concepts, sources, and knowledge gaps (Mays et al., 2001). Therefore, the scoping review method is particularly suitable for gathering existing research on the specific concept of counseling competency. In this study, this method is used to explore existing evidence, clarify counseling competencies, and identify potential research gaps in the field.

### 1.4. The Objectives of Current Study

In summary, the present study employed a scoping review methodology to identify the requisite competencies for counselors. This review encompassed three primary objectives: (1) assessing the adequacy of available empirical research evidence on this topic, (2) identifying the core competencies for counselors and refining the definition of each competency, and (3) providing a comprehensive framework to synthesize the varied competencies.

## 2. Methods

This scoping review was conducted following the guidelines of the Preferred Reporting Items for Systematic Reviews and Meta-Analysis Extension for Scoping Reviews (PRISMA-ScR) checklist (Tricco et al., 2018). The final protocol of this study was registered with the Open Science Framework on 18 June 2023.

### 2.1. Search Strategy

A comprehensive literature search was performed to retrieve articles from five databases up to 28 March 2023. Specifically, the databases searched included Web of Science, PsycINFO, PubMed, WANFANG, and China National Knowledge Infrastructure (CNKI). The latter two databases were specifically utilized for identifying Chinese-language articles. Two categories of keywords, namely, “counselors” and “competencies”, were used to search potential articles. The terms related to counselors were “therapist*”, “counselor*”, and “psychotherapist*”. The terms related to competency were “professional competenc*”, “capabilit*”, and “therapeutic competenc*”. Note that the asterisk symbol “*” serves as a wildcard character in this context. The selection of the final terms resulted from a balance between the pursuit of comprehensiveness and the necessity to maintain relevance. This process was undertaken during the preliminary search phase. The researchers logged in to the websites and used their advanced search functions, which support the use of various search strategies combining different terms. More detail on the search strategy for each of the five databases is available in the Supplementary Materials. Following the database searches, the reference lists of the identified literature were systematically reviewed and analyzed.

### 2.2. Eligibility Criteria

Based on the objectives of this study, a set of eligibility criteria was established to guide the screening process. The inclusion criteria were as follows: (1) The research topic is directly relevant to counseling competencies; (2) the participants are counseling practitioners; (3) the article is written in either Chinese or English. Given that the research team is Chinese, we included articles not only in English but also in Chinese; and (4) the studies are of an empirical nature, with a specific study design and original datasets, thereby ensuring a rigorous and empirical-evidence-based analysis of counseling competencies.

The following situations led to the exclusion of studies: (1) Studies focusing on counselors in specialized fields, such as sports, schools, or marriage and family, were excluded to maintain a broader perspective for competencies across diverse contexts; (2) studies exclusively focusing on specific counseling schools, such as CBT, were excluded, as we focused on universal and common competencies applicable to all counselors; (3) research unrelated to competencies or solely focused on one specific skill, such as empathy, was excluded considering the main focus of this study; (4) studies for which the full text was not accessible were excluded; (5) reviews, conference literature, and protocols were also excluded.

### 2.3. Screening Strategy

All identified articles were first imported into Endnote 20, and then duplicate entries were eliminated. Subsequently, the selected articles were imported into Rayyan (https://www.rayyan.ai/, accessed on 5 April 2023), an internet-based platform designed specifically for conducting systematic reviews. Following the predefined inclusion and exclusion criteria, the articles were first screened based on their titles and abstracts, and then had a more detailed examination by review of the full texts.

This screening procedure was carried out by the first three authors working in pairs, ensuring that each article went through independent assessment and data extraction by two researchers. The intraclass correlation coefficients (ICCs) among raters in the systematic review’s two phases (abstract screening and full-text inclusion) were 0.95–0.99, indicating high reliability. Coding conflicts centered on (1) studies on specific counseling domains (e.g., CBT, school-based); (2) studies involving scale development/revisions; (3) research on specific client populations (e.g., LGBTQ+); and (4) cultural competence studies. Any discrepancies that arose during the screening process were addressed through collective discussion among the team members. In situations where consensus could not be reached, the expertise of the last author, an experienced professor with more than 20 years of counseling experience, was sought for guidance. With the supervision of the expert, discussions were conducted until a complete consensus was achieved, thereby ensuring a rigorous and meticulous approach to the screening process.

### 2.4. Data Extraction and Analysis

Qualitative data from the selected literature was recorded using Microsoft Excel 2019. The data-charting form was created and refined through the coding of data from three eligible studies. The final iteration of the extracted entries included the title, first author, publication year, study location, publication type, journal, sample size, sample source, research method, and key findings. Then, the first three authors collaborated in pairs to independently complete the data extraction for each eligible study using Rayyan. In cases of disagreement over item extraction, the guidance of the expert was sought and discussion between the research team was conducted to reach a consensus.

To structurally organize the findings and enhance the quality of this review, we followed the Grounded Theory method (Glaser & Strauss, 1999/2017). Grounded theory has been widely applied to code qualitative data and identify core concepts that reflect social phenomena. The coding process involved three stages. First, in the open coding phase, all competencies mentioned in the included articles were extracted, and original expressions from the articles were retained. Next, in the axial coding phase, identical and recurring terms were grouped together, primarily based on the definitions of the competencies provided in the original articles. For example, “Passion for counseling” and “Enthusiasm” were grouped into the passion competency. Finally, in the substantive coding phase, the competencies were categorized into core categories based on their shared characteristics. For instance, “Humanistic Knowledge” and “Professional Knowledge” were categorized into the knowledge category.

## 3. Results

### 3.1. Article Selection

The initial search yielded a total of 4174 articles, out of which 941 duplicates were eliminated. Subsequently, 3126 articles were excluded during the screening of titles and abstracts, and an additional 92 articles were excluded after a thorough assessment of the full-text articles for eligibility.

The inclusion and exclusion criteria were strictly followed. First, the majority of articles (n = 1927 + 49) were excluded because their research samples did not focus on psychotherapists or psychological counselors. Instead, they included therapists or counselors whose primary focus was on other aspects (e.g., physical therapists, speech therapists, occupational therapists, genetic counselors, rehabilitation counselors, peer counselors), those utilizing specialized skills beyond typical counseling conversations (e.g., art therapists, music therapists, game therapists, recreational therapists), or individuals such as supervisors for psychotherapists, psychiatrists, psychology teachers, pastors, or social workers. Second, another reason for excluding a large portion of articles (n = 1015 + 24) was their focus on specific aspects of counselors’ competencies (such as empathy, skills in asking open questions, and non-verbal communication) or specific conditions (such as ethical dilemmas and the need for supervision), rather than adopting a holistic perspective. Third, a number of publications (n = 110 + 4) were excluded because they were not empirical studies with specific research designs and participants, but rather other types of publications such as reviews, book chapters, and letters. Fourth, another reason for excluding articles (n = 74 + 6) was that they were written in languages other than English or Chinese, such as Korean, German, and Russian, which are beyond the authors’ ability to comprehend. Lastly, nine articles were excluded because their full texts could not be accessed.

**Figure 1:**
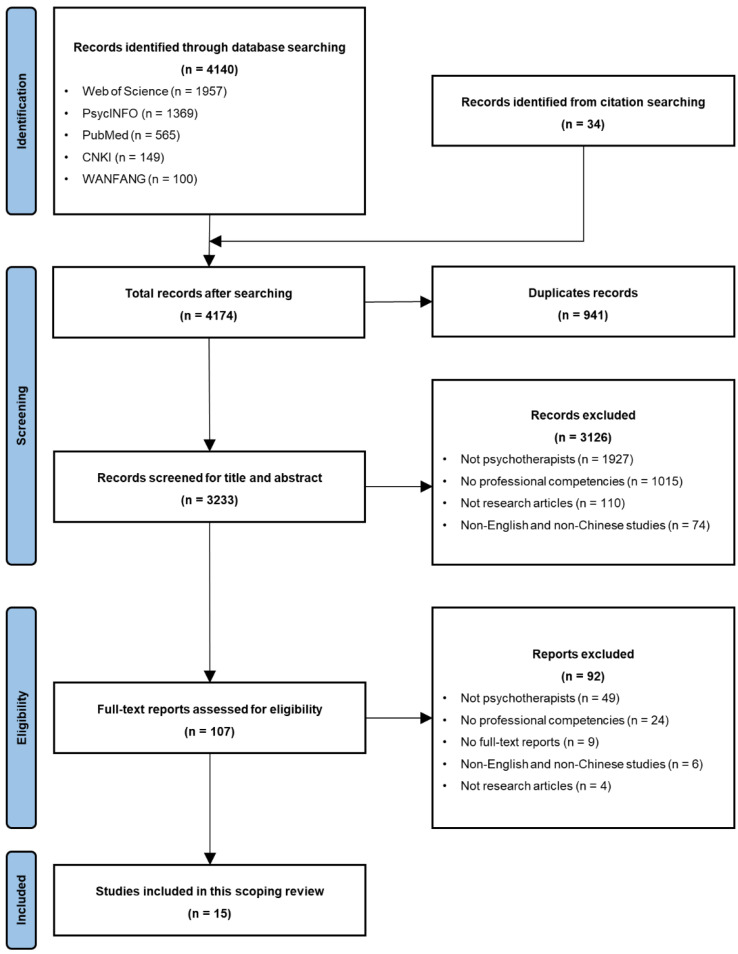
Flow chart of PRISMA process for a scoping review.

Therefore, a total of 15 articles were ultimately included in this review (see the flow chart of the process in Figure 1).

### 3.2. Article Characteristics

Table 1 presents a summary of the characteristics of the included articles. As for the study location, the majority of the studies (n = 12) originated in the United States (n = 6) and China (n = 6), while the remaining three studies were conducted in South Africa, Germany, and Italy. Of the included studies, nine were written in English and six in Chinese. As for the sample source, among the 15 studies, six involved participants from diverse backgrounds, including faculty members, counselors in training, counselor educators, and non-specialists. Faculty members and counselors in training were the most frequently represented participant groups, appearing in over half of the studies, while non-specialists were the focus of two studies. As for the research method, survey-based methods were mostly utilized (n = 12), followed by interview-based methods (n = 10). Mixed-method data (n = 7) were the most frequently reported, followed by quantitative data (n = 5) and qualitative data (n = 3).

## 4. Synthesis of Results

### 4.1. Core Competencies for Counselors

From the 15 included studies, a total of 122 competencies were initially identified. Through axial coding, 30 core competencies were summarized. The specific core competencies include humanistic knowledge, professional knowledge, communication, assessment, intervention, influence, empathy, awareness, thinking, multicultural skill, application of theory, controllability, relationship building, consistency, self-growth, interest, respect, patience, altruism, sincerity, acceptance, passion, flexibility, warmth, understanding, emotional sensitivity, openness, responsibility, integrity, and self-confidence. The respective definitions pertaining to every core competency are exhibited in Table 2. The sources supporting the definitions of each core competency are provided. Additionally, the articles that examine the competencies are also presented for each core competency.

### 4.2. Categories of the Core Competencies

These competencies were further categorized into four overarching categories: Attitude, knowledge, personality, and skill and ability. Based on previous studies, (1) attitude is defined as a relatively enduring organization of beliefs, feelings, and behavioral tendencies toward socially significant objects, groups, events, or symbols (Hogg & Vaughan, 2005), including counseling competencies such as interest, respect, patience, altruism, sincerity, etc.; (2) personality is a particular feature or quality of a person, animal, or other unit of interest, especially any of the enduring qualities or traits that define an individual’s nature or personality in relation to others (VandenBos, 2007), including counseling competencies such as passion, flexibility, warmth, etc.; (3) knowledge is defined as awareness, information, or understanding of facts, rules, principles, guidelines, concepts, theories, or processes needed to succeed in a specific content area (Marrelli, 1998; Spencer & Spencer, 1993), including counseling competencies such as humanistic knowledge, and professional knowledge; and (4) skill and ability is defined as a cognitive or physical capacity to perform tasks with a successful outcome (Hoge et al., 2005), including counseling competencies such as communication, assessment, intervention, influence, etc.

### 4.3. Frequency of Competencies in Included Studies

**Figure 2:**
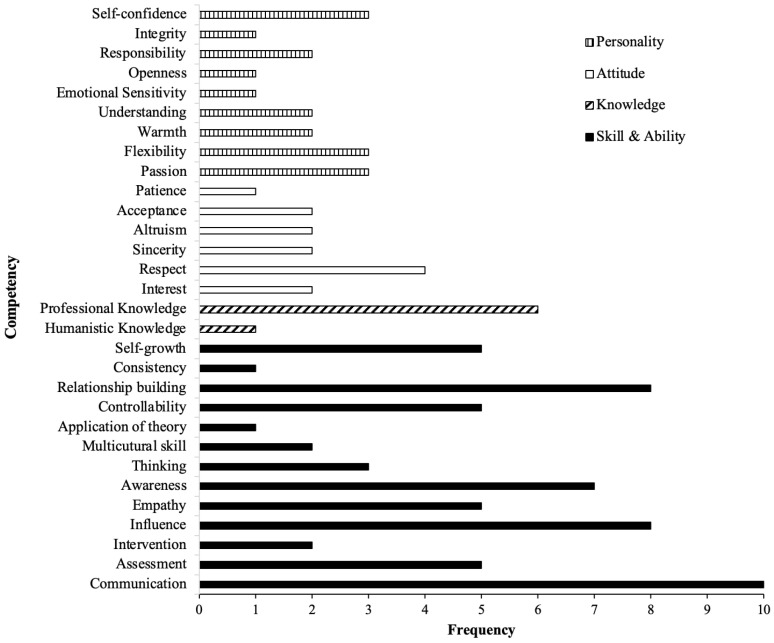
Frequency of each competency in included articles (n = 15).

The frequencies of 30 competencies observed in the included articles are shown in Figure 2. Communication emerged as the most emphasized competency, being discussed in 10 articles. Relationship building and influence, with equal frequencies, were discussed at the second highest rate. On the other hand, integrity, openness, emotional sensitivity, patience, humanistic knowledge, consistency, and application of theory received the least attention, being mentioned in only one of the reviewed articles.

## 5. Discussion

### 5.1. Main Findings

This scoping review synthesized the current evidence for counseling competency and utilized a three-code procedure based on the Grounded Theory method to extract core competencies and their categories. With the strict inclusion and exclusion criteria for pieces of literature, only 15 qualified articles were found to report findings about counseling competency from a well-rounded perspective. Among these studies, 30 core competencies were extracted with their definitions explicitly demonstrated, contributing to well understanding of each competency. Furthermore, these competencies were categorized into four themes: attitude, personality, knowledge, and skill and ability, providing a sensible framework for organizing multi-structure core competencies. This work could be of great significance not only for counseling psychologists to explore the concept of counseling competency, but also for counseling training and related organizations to foster high-quality counseling services.

### 5.2. Characteristics of Existing Evidence for Counseling Competency

As for the quantity of current literature on counseling competency, it is notable that the existing journal publications on this topic were rather limited, with only 15 studies found to meet our criteria. One of the reasons for the lack of related studies might lie in the nature of counseling competency being too broad to be investigated. Compared with evidence-based empirical studies, we found more book publications providing narrative and detailed discussions on the understanding and training on counseling competency (Ali & Sichel, 2014; Brinson et al., 2008; Sperry & Sperry, 2023). Another reason for the limited number of studies included is that only studies from a broad perspective were considered. It is important to note that more studies on counseling competency are available from some specific viewpoints. For instance, Köse (2019) emphasized the need for program evaluation competencies in school counseling, particularly in technical skills and interpersonal relationships. Gang and Xi (2024) further underscored the impact of counselor competencies on job performance, particularly in crisis intervention, career counseling, active listening, and interpersonal skills. Therefore, the screening provided the first finding suggesting the great lack of empirical studies on counseling competency, calling for more investigations to provide more information and further exploration.

Further analysis of the specific characteristics of the studies showed the uneven location distribution of the studies and the variety of the study designs. Firstly, we found most of them from the United States (n = 6) and China (n = 6), while articles were rarely found in other countries. In addition, among the included studies, we found distinct research objects and methods between studies from the US and those from China. Specifically, studies from the United States, like those by Eriksen and McAuliffe (2003) and Torres-Rivera et al. (2002), prioritized the development of competency scales in counselor education. Conversely, Chinese research focused on competency models, indicating a cultural emphasis on holistic counselor development. Secondly, as for the sample source, the current studies mainly concentrated on counselors themselves. However, as Kaslow et al. (2007) suggested, assessment of competencies necessitates a multi-source approach. This means that opinions from counselors as well as their clients should be comprehensively considered. At least in this scoping review, we still did not identify any study using such a multi-source approach. Thirdly, as for the research methods, interviews and surveys were the most commonly employed in these studies. Structured interviews, like those in Eriksen and McAuliffe (2003), ensured consistency in competency assessment, while semi-structured formats offered flexibility to explore nuances (Kühne et al., 2021; Spedding et al., 2022). Surveys leveraged both established scales, such as the Counseling Competencies Scale by Lambie et al. (2018), and study-specific instruments like the QACP by Settanni et al. (2022), to evaluate a range of competencies. These methods provided a comprehensive toolkit for assessing counselor capabilities, from standardized to context-specific evaluations, reflecting the multifaceted nature of competency measurement.

### 5.3. Definitions and Categories of Core Counseling Competencies

The most important result of this scoping review was the list and definition of the 30 core competencies we have extracted. Admittedly, several standards documents on counseling competency have been established. For example, the European Training Standards Committee (EAP, 2013) defined and established 13 categories of professional competencies for a European psychotherapist, including the psychotherapeutic relationship, completion and evaluation, ethics, cultural sensitivities, etc. Nevertheless, the scoping review is still of great significance, since we provide a new perspective with a specific emphasis on empirical studies worldwide. Moreover, we further clarified the definitions for each counseling competency based on extensive references. The comprehensive presentation of these definitions enhances conceptual understanding and fosters the construction of theoretical frameworks. It also holds practical implications for guiding the application of these competencies in real-world settings. Among the myriad studies conducted, this scoping review stands as the inaugural systematic overview to provide a complete set of definitions. It bridges the gap between theoretical constructs and their practical application, enriching the field of psychological counseling with a well-defined foundation for professional development and practice.

Apart from the definitions, we further constructed four categories for the core competencies—attitude, personality, knowledge, and skill and ability. First of all, as a well-clarified structured model, this result furnishes a coherent framework for the conceptualization of counseling competencies. This model introduces a hierarchical and systematic approach to training and practice, contributing to the sense of control and efficacy in the conduction of training. Since the model simplified the thirty core competencies into four categories, it also reduces the cognitive load on remembering them. For a specific category, the various competencies within it might share a stronger synergistic promoting effect. For example, two typical competencies within the skill and ability category, communication and cultural skills, have shown a significantly positive correlation in previous studies (e.g., Setiyowati et al., 2019). As for the four differentiated categories, the model could inform us of the application of tailored training methods best suited to each category. For instance, the knowledge domain can be effectively addressed through theoretical instruction and case study analysis (Kealy et al., 2022), while the skill and ability domain benefits from more interactive and experiential approaches such as role playing and hands-on practice (Hill & Norcross, 2023). Further investigation across and within the diverse categories is warranted to provide a more comprehensive understanding and meaningful implications for practical application.

### 5.4. Limitations

Some limitations of this study should be noted. First, although we aimed to adopt a cross-cultural perspective, the publications included in this scoping review are predominantly from the US and China, with a few from South Africa, Germany, and Italy, and there is a lack of studies from other countries. Meanwhile, only publications in English and Chinese were included, which may have resulted in the exclusion of data from other linguistic sources (e.g., Korean, German, Russian). Therefore, caution should be exercised when generalizing the findings of this study to counselors in different countries. Second, the core competencies are provided with definitions and references but lack real-life examples to further illustrate their application, which is beyond the scope of the current study. However, it would be worthwhile for future studies to provide such demonstrations and encourage practitioners to relate these competencies to their previous experiences. Third, the use of the “Rayyan” tool might reduce the authors’ sensitivity during article screening, potentially leading to the exclusion of some relevant articles. However, during the initial screening stage, we ensured that each article’s title and abstract were reviewed.

### 5.5. Implications

Despite the limitations, the current scoping review on counseling competencies offers significant theoretical and practical contributions.

First, this scoping review identified 30 core competencies for psychological counselors. Each of these core competencies was provided with clear definitions and references. These results could allow researchers and practitioners to understand psychological counselor competencies in a more comprehensive and detailed way. For researchers, these findings lay a foundation for future studies in this field. For instance, identifying specific competencies could promote further exploration of topics such as the dynamic interactions between competencies and their relative importance in different contexts. For practitioners, these competency descriptions can serve as a practical guide, supporting the training, selection, and professional development of counselors. For instance, the list of 30 core competencies could function as a checklist for counselors to review before each session, ensuring they are well prepared.

Second, a four-dimensional framework—attitude, personality, knowledge, and skill and ability—has been proposed. This framework is easy to understand and aligns with findings that may have been suggested in books or classes. Notably, this scoping review derived its results systematically and reproducibly, grounded in empirical studies. This approach ensures that the findings are relatively objective, offering stronger evidence to support the adoption of this framework in future studies.

Third, this scoping review examined the adequacy of existing literature on psychological counselor competencies and revealed the scarcity of related empirical research. The identification of this research gap represents a crucial step toward stimulating further empirical exploration, particularly from a holistic perspective of counselor competencies.

## 6. Conclusions

This scoping review screened, mapped, and synthesized the existing literature on counseling competency. First, the findings highlight the lack of empirical studies with rigorous research designs on the topic of counseling competencies. Second, within the 15 articles included in this scoping review, a total of thirty core competencies was identified, such as humanistic knowledge, assessment, intervention, empathy, awareness, flexibility, warmth, understanding, emotional sensitivity, among others. The respective definitions for each core competency are provided, supported by relevant references. Third, a four-dimensional framework—attitude, personality, knowledge, and skill and ability—has been proposed. These findings could serve as valuable resources for further research on this construct and for enhancing counseling training.

## Figures and Tables

**Table 1:** Characteristics of included articles.

Author (Year)	Country	Study Design	Sample	Instrument(s)	Competencies
Chen (2022)	China	Mixed methods (literature review, interview, and survey)	20 psychological counselors, 128 psychological counselors in training	Counselor Competency Self-Assessment Scale	Self-growth, helping people, passion for counseling, self-confidence, enthusiasm, affinity, empathy, comprehension, insight, expression, problem diagnosis, relationship building, problem solving, psychological assessment, professional knowledge, humanistic knowledge, interest in reading, growth learning, responsibility, confidentiality, values, integrity, listening, patience, acceptance
Eriksen and McAuliffe (2003)	United States	Quantitative (interview and survey)	24 educators for psychological counselors, 29 psychological counselors in training	Counseling Skills Scale	Interest, exploration, deepen, change, relationship, management
He (2020)	China	Qualitative (literature review, observation, and interview)	7 psychological counselors in training	Behavioral Event Interview Guide	Interests of clients are primary, equality, consistency, objective presentation, sincerity, admit your own shortcomings, insight, analytical thinking, empathy, awareness, acceptance, ethical codes, professional knowledge of psychological consultation
Kühne et al. (2021)	Germany	Mixed methods (interview and survey)	375 non-specialists from general population	Two open-ended questions, a scale	Work-related principles, professionalism, personality characteristics, caring communication, empathy and understanding
Lambie et al. (2018)	United States	Quantitative (survey)	356 psychological counselors in training, 24 educators for psychological counselors	Counseling Competencies Scale (Swank et al., 2012)	Counseling skills and therapeutic conditions, counseling dispositions and behaviors
Li (2012)	China	Mixed methods (interview, literature review, and survey)	141 psychological counselors	Self-rating Psychological Competence Scale	Occupation motivation, personality, interpersonal communication, knowledge, professional skills
Luo (2008)	China	Qualitative (interview)	34 psychological counselors	Spencer and Spencer (1993)’s universal competence model of service providers	Influence, compathy, sincerity, flexibility, controllability, self-confidence, analytical thought, insight/awareness, relationship building, learning
Peterson and Bry (1980)	United States	Mix methods (interview and survey)	14 psychological counselors, 191 educators for psychological counselors	Professional Competence Inventory	Professional responsibility, interpersonal warmth, intelligence, experience
Swank et al. (2012)	United States	Quantitative (survey)	188 psychological counselors in training	Counseling Competencies Scale	Professional behaviors, counseling relationship, counseling skills, assessment and application, professional dispositions
Settanni et al. (2022)	Italy	Quantitative (survey)	778 psychological counselors in training and psychological counselors	Self-Assessment Questionnaire of Psychotherapist’s Competencies	Assessment and case formulation, therapeutic relationship, implementation of the intervention, evaluation and conclusion of therapy, ethics and cultural sensitivity
Spedding et al. (2022)	South Africa	Mixed methods (interview and survey)	40 psychological counselors, 4 non-specialists	Enhancing Assessment of Common Therapeutic factors for South Africa	Communication, emotional engagement, process and intervention, counselor qualities and characteristics
Torres-Rivera et al. (2002)	United States	Quantitative (survey)	248 psychological counselors in training	Counselor Skills Personal Development Rating Form (Wilbur et al., 1994)	Emotional sensitivity, basic listening skills, multicultural skills, influencing skills
White (1980)	United States	Mixed methods (interview and survey)	705 educators for psychological counselors	Questionnaires developed from the interview	Personal development, research and professional activities, behavioral strategies in counseling, application of counseling theory, class participation, relationship strategies in counseling, collaboration, efficiency, respect for individuality, flexibility
Wu (2010)	China	Qualitative (interview)	20 psychological counselors	Coding manual for psychological counselors’ competency research	Flexibility, influence, experience, basic attitude to set up a relationship with clients, interpersonal understanding, self-awareness, self-control, mental health, professional knowledge and skills, altruism, respect of clients, ability to training others, language ability, openness
Xiang (2007)	China	Mixed methods (interview and survey)	251 psychological counselors	Competence of Psychotherapist Self-Rating Form	Self-retrospection, empathy, self-confidence, professional expertise, conceptual thinking, linguistic expressing, respecting others, developing others, impact and influence, self-control

**Table 2:** The definition of each competency.

Category	Competency	Definition	Included Articles That Mentioned This Competency	References for Definition
Knowledge	Humanistic knowledge	Knowledge of different cultures, languages, regions of life, political and economic patterns, as well as knowledge of different subjects and non-subjects.	Chen (2022)	He et al. (2011)
	Professional Knowledge	Proficient in work-related knowledge and motivated to expand, use, and disseminate work-related knowledge to others.	He (2020); Wu (2010); Xiang (2007)	Xiang (2007)
Skill and ability	Communication	Be good listeners with good interpersonal communication and adaptability skills, maintain eye contact, use goal-oriented questions, and enable their clients to talk for most of the session.	Kühne et al. (2021); Li (2012); Spedding et al. (2022)	Kühne et al. (2021); Li (2012)
	Assessment	Assessment and diagnosis of problems, capabilities, and issues associated with individuals, groups, and/or organizations.	Settanni et al. (2022); Swank et al. (2012); Chen (2022)	Fouad et al. (2009)
	Intervention	Interventions designed to alleviate suffering and to promote the health and well-being of individuals, groups, and/or organizations.	Settanni et al. (2022); Spedding et al. (2022)	Fouad et al. (2009)
	Influence	Influence to present a specific impact or influence on others without relying on power by virtue of one’s own personal qualities.	Luo (2008); Wu (2010); Xiang (2007)	Luo (2008); Xiang (2007)
	Empathy	Be empathic, understanding, compassionate, and caring.	Xiang (2007); Chen (2022); Kühne et al. (2021); He (2020); Luo (2008)	Xiang (2007); Luo (2008); Kühne et al. (2021)
	Awareness	The ability to perceive, understand, and anticipate the emotions and thoughts of others and oneself, and most importantly, to discover the root causes behind problems.	He (2020); Wu (2010); Luo (2008)	Luo (2008)
	Thinking	Using assembling pieces and looking at the big picture to understand a situation or problem, including identifying links or patterns between situations with no apparent relationship, and identifying key or underlying issues in complex situations.	Xiang (2007); He (2020); Luo (2008)	Xiang (2007); Luo (2008)
	Multicultural skill	The ability of counselors-in-training to recognize and deal with issues related to diversity, racism, and prejudice that affect the counseling relationship during a counseling session.	Torres-Rivera et al. (2002)	Torres-Rivera et al. (2002)
	Application of theory	Used theory to analyze behavior and counselor–client interactions, discussed with supervisor. Encouraged clients to share feelings and thoughts, acknowledged them, and paraphrased clearly. Shared reflections in seminars, adjusted proximity and eye contact during sessions, and recognized personal biases. Supported peers by reflecting their emotions.	White (1980)	White (1980)
	Controllability	Active control of the direction and content of counseling or passive following of the visitor in counseling.	Luo (2008)	Luo (2008)
	Relationship building	To create and consistently engage in a caring, warm, safe atmosphere for visitors, so that visitors trust the counselor, are willing to expose themselves, take the initiative to explore, and work with the counselor to solve their own problems.	Eriksen and McAuliffe (2003); Luo (2008); Wu (2010); Settanni et al. (2022); White (1980); Swank et al. (2012); Chen (2022)	Eriksen and McAuliffe (2003); Luo (2008); White (1980)
	Consistency	Consistency is a state of being stable and not susceptible to change. It consists of three levels of meaning: the first is consistency of thought with thought, the second is consistency of thought with action, and the third is consistency of action with action.	He (2020)	He (2020)
	Self-growth	Continually consults information, discusses with peers, supervises, self-summarizes, and seeks feedback on the impact of their behavior on others in order to achieve self-growth.	White (1980); Chen (2022); Luo (2008)	White (1980); Luo (2008)
Attitude	Interest	Interest requires love and respect for one’s own profession.	Chen (2022); Eriksen and McAuliffe (2003); He (2020)	Liang (2009)
	Respect	Respect requires the counselor to allow and respect others to have feelings and thoughts different from their own. In the consultation process, the counselor should clarify the roles and responsibilities of both parties, and adjust the consultation methods and styles according to the characteristics of different visitors.	White (1980); Wu (2010); Xiang (2007); Spedding et al. (2022)	White (1980)
	Patience	Patience has been defined as warmly as “a nurturing capacity”, as sterile as a long-term reward response, and as vaguely as simply the behavioral act of waiting.	Chen (2022)	Lavelock (2016)
	Altruism	A moral norm [which] implies certain social expectations of helping others in different social contexts	Wu (2010)	Bykov (2017); Pfattheicher et al. (2022)
	Sincerity	In counseling, the counselor should appear as the “real me”, without defensive camouflage, not hiding behind a professional role, not playing a role, but consistent with the outside and the inside, and truly and credibly in the relationship with the help seeker.	He (2020); Luo (2008)	Luo (2008)
	Acceptance	The active and aware embrace of either internal experiences or others’ experiences without changing their frequency or form.	Chen (2022); He (2020)	Pakenham (2015)
Personality	Passion	Passion for work is defined as a strong inclination toward an activity that people like, that they find important, that is self-defining, and in which they invest time and energy.	Chen (2022)	Birkeland and Nerstad (2016)
	Flexibility	Flexibility is manifested in that the counselor can create different atmospheres and use different methods according to others’ feelings and problems when facing different clients.	Luo (2008); White (1980); Wu (2010); Lambie et al. (2018); Spedding et al. (2022)	Luo (2008); White (1980)
	Warmth	Warmth always relates to perceived intent, including friendliness, helpfulness, sincerity, trustworthiness, and morality.	Peterson and Bry (1980); Spedding et al. (2022)	Fiske et al. (2007)
	Understanding	Understanding involves understanding the client’s symptoms, behaviors, and difficulties, understanding their subjective position, and expressing empathy.	Kühne et al. (2021); Wu (2010)	The European Training Standards Committee (EAP, 2013)
	Emotional Sensitivity	Emotional sensitivity can be defined as a lower threshold to detect or respond to emotional stimuli, or a higher probability of experiencing stimuli as emotional.	Torres-Rivera et al. (2002)	van Zutphen et al. (2015)
	Openness	An appreciation for spiritual diversity	Wu (2010); Peterson and Bry (1980); Lambie et al. (2018)	Barron (2012)
	Responsibility	The counselor should have a serious and responsible attitude towards clients and their work, and can bear the pressure and problems in work. For the sake of clients or self-growth, they should dare to take certain risks and bear the possible consequences.	Chen (2022); Peterson and Bry (1980)	R. Q. Wang (2008)
	Integrity	Integrity is a deeply personal phenomenon, also the correlation between actions and beliefs, principles, or convictions on the other.	Chen (2022); Peterson and Bry (1980)	LaSala (2009)
	Self-Confidence	A person believes in their ability to perform a task, including the confidence shown in dealing with difficult circumstances, making decisions, or facing challenges.	Chen (2022); Luo (2008) Xiang (2007); Spedding et al. (2022)	Luo (2008)

## Data Availability

All data and materials used have been provided or illustrated in the main text.

## Supplementary Materials

The following supporting information can be downloaded at: https://www.mdpi.com/article/10.3390/bs15020147/s1.
