# Supplementary material for: Core Competencies for Psychological Counselors: A Scoping Review
Source: Behav Sci (Basel). 2025 Jan 29;15(2):147. doi: 10.3390/bs15020147 (PMC11851845; doi:10.3390/bs15020147)
Supplement: Supplementary file 1 [file behavsci-15-00147-s001.zip › behavsci-3406819-supplementary.pdf]

Supplementary Material: Literature Review Search Records

This document provides a detailed account of the search strategies and outcomes of the literature review conducted for this study. The following table summarizes the search parameters and results across various databases.

Database	Date	Search Strategy	Total Results	Document Types	Notes
CNKI	18 Mar 2023	(SU %= "psychological counselor" + "psychotherapist" + "counselor") AND (SU %= "competence" + "competency" + "capability" + "core ability" + "competent capacity" + "competent quality")	149	Academic Journals, Theses, Conference Papers	Bilingual (Chinese and English) search; topic-based search strategy
Wanfang	18 Mar 2023	(Topic: "psychological counselor" OR "psychotherapist" OR "counselor") AND (Topic: "competence" OR "competency" OR "capability" OR "core ability" OR "competent capacity" OR "competent quality")	100	Academic Journals, Theses, Conference Papers	Bilingual search; topic-based search strategy
WOS	28 Mar 2023	TS=(("therapist*" OR "counselor*" OR "psychotherapist*") AND ("professional competenc*" OR "capabilit*" OR "therapeutic competenc*"))	1957	Not specified	Topic-based search strategy; comprehensive search without document type restrictions
PsycINFO	27 Mar 2023	(SU("therapist*" OR "counselor*" OR "psychotherapist*")) AND (SU("professional competenc*" OR "capabilit*" OR "therapeutic competenc*"))	1369	Academic Theory Journals, Research Reports, Electronic Journal Collections	Topic-based search strategy; focused on psychological and behavioral sciences

PubMed	27 Mar 2023	(("therapist*"[Title/Abstract] OR "counselor*"[Title/Abstract] OR "psychotherapist*"[Title/Abstract]) AND ("professional competenc*"[Title/Abstract] OR "capabilit*"[Title/Abstract] OR "therapeutic competenc*"[Title/Abstract]))	565	Not specified	Title/Abstract-based search strategy; relevant to medical and life sciences

Notes:

[1] The search strategies were designed to capture a broad range of literature relevant to the study's focus on the professional competencies of psychological counselors.

[2] Bilingual search strategies were employed to include both Chinese and English-language literature.

[3] SU: Subject terms or keywords used in the search strategy.

[4] TS: Topic search, indicating a search across the entire text of the documents.

[5] Topic: Keywords related to the subject area of the search.

[6] Title/Abstract: Search limited to the titles and abstracts of the documents.

This supplementary material is provided to ensure transparency in the literature review process and to demonstrate the comprehensive nature of the search strategies employed.
